# Supplementary material for: Ot2Rec: A semi-automatic, extensible, multi-software tomographic reconstruction workflow
Source: Biol Imaging. 2023 Mar 29;3:e10. doi: 10.1017/S2633903X23000107 (PMC10936412; doi:10.1017/S2633903X23000107)
Supplement: Supplementary file 1 [file S2633903X23000107sup001.docx]

**Supplementary Information**

**Ot2Rec: A Semi-Automatic, Extensible, Multi-Software Tomographic Reconstruction Workflow**

Neville B.-y. Yee^1^, Elaine M. L. Ho^1^, Win Tun^1,2^, Jake L. R. Smith^3,4^, Maud Dumoux^3^, Michael Grange^3,4^, Michele C. Darrow^1,5^, and Mark Basham^1,6^

^1^Artificial Intelligence & Informatics, Rosalind Franklin Institute, Harwell Campus, Didcot, OX11 0FA, UK

^2^Faculty of Medical Sciences, Newcastle University, Newcastle upon Tyne, NE1 7RU, UK

^3^Structural Biology, Rosalind Franklin Institute, Harwell Campus, Didcot, OX11 0FA, UK

^4^Division of Structural Biology, Wellcome Centre for Human Genetics, University of Oxford, Oxford, OX3 7BN, UK

^5^SPT Labtech, Melbourn Science Park, Cambridge Road, Melbourn, SG8 6HB, UK

^6^Diamond Light Source Ltd., Diamond House, Harwell Campus, Didcot, OX11 0DE, UK

Correspondence to be addressed to: [Neville.Yee@rfi.ac.uk](mailto:Neville.Yee@rfi.ac.uk), [Mark.Basham@rfi.ac.uk](mailto:Mark.Basham@rfi.ac.uk).

Contents:

| Ot2Rec Report for Case Study 1 | 1 |
| --- | --- |
| Ot2Rec Report for Case Study 2 | 11 |
| Ot2Rec Report for Case Study 3 – IMOD | 21 |
| Ot2Rec Report for Case Study 3 – AreTomo | 50 |
| Table S1: Configuration parameters for Case Study 1 | 70 |
| Table S2: Configuration parameters for Case Study 2 | 71 |
| Table S3: Configuration parameters for Case Study 3 | 72 |
| Guide on writing plugins for Ot2Rec | 73 |
| Tutorial to reproduce Case Study 1 in Ot2Rec | 81 |
| Example: Using Ot2Rec in Jupyter Notebooks | 88 |
| Movie S1: Video of 3D reconstructions from the various workflows in Case Study 3 | 93 |

Ot2Rec Report for Case Study 1

**Workflow diagram**

# Motion correction

Motion correction has been performed using MotionCor2.

Please cite:

1. Zheng, S., Palovcak, E., Armache, JP. et al. MotionCor2: anisotropic correction of beam-induced motion for improved cryo-electron microscopy. *Nat Methods* **14**, 331–332 (2017).

## Summary of shifts for each tilt series

**mean	std	25%	50%	75%**

**TiltSeries**

**1**	0.597280	0.645662	0.158114	0.460000	0.806598

| **2** | 0.935666 | 1.117344 | 0.228473 | 0.664831 | 1.245311 |
| --- | --- | --- | --- | --- | --- |
| **3** | 0.506196 | 0.512616 | 0.152315 | 0.402244 | 0.698140 |
| **4** | 0.529967 | 0.593529 | 0.140357 | 0.410122 | 0.696348 |
| **5** | 0.671132 | 0.738262 | 0.177200 | 0.500100 | 0.882553 |
| **6** | 0.501673 | 0.482156 | 0.155242 | 0.433820 | 0.705762 |
| **7** | 0.423647 | 0.391669 | 0.138924 | 0.368782 | 0.604070 |
| **8** | 0.568803 | 0.542346 | 0.170880 | 0.481041 | 0.798812 |
| **9** | 0.466622 | 0.468324 | 0.136015 | 0.390512 | 0.638905 |
| **10** | 0.468918 | 0.466595 | 0.142127 | 0.399625 | 0.654905 |
| **11** | 0.552081 | 0.562856 | 0.161245 | 0.456946 | 0.763217 |
| **12** | 0.529845 | 0.521374 | 0.166433 | 0.452769 | 0.754321 |
| **13** | 0.669184 | 0.717781 | 0.182483 | 0.512640 | 0.906091 |
| **14** | 0.634099 | 0.676261 | 0.176918 | 0.494975 | 0.857030 |
| **15** | 0.695593 | 0.703848 | 0.205913 | 0.576281 | 0.968762 |
| **16** | 0.489171 | 0.458651 | 0.152971 | 0.417732 | 0.683520 |
| **17** | 0.417093 | 0.367335 | 0.130384 | 0.377359 | 0.610000 |

Out[12]: Text(0.5, 1.0, 'Euclidean Shift Means +/- Standard Deviation')

# IMOD (general)

IMOD has been used in this image processing operation.

Please cite:

1. James R. Kremer, David N. Mastronarde, J.Richard McIntosh.

Computer Visualization of Three-Dimensional Image Data Using IMOD, *Journal of Structural Biology*

**116(1)**:71-76 (1996)

2. Mastronarde DN, Held SR.

Automated tilt series alignment and tomographic reconstruction in IMOD, *Journal of Structural Biology* **197(2)**:102-113 (2017)

## Tilt-series Alignment (IMOD)

**Shifts between patches in A**

Taken from .xf file

Out[13]:	**Tilt series	Mean shift (A)	Shift s.d. (A)**

**0**	1	12.740374	15.802750

| **1** | 2 | 19.183049 | 11.365527 |
| --- | --- | --- | --- |
| **2** | 3 | 38.332763 | 13.413515 |
| **3** | 4 | 26.860351 | 10.567297 |
| **4** | 5 | 17.211399 | 4.665976 |
| **5** | 6 | 23.029534 | 6.364917 |
| **6** | 7 | 24.225329 | 10.100222 |
| **7** | 8 | 29.900118 | 11.788393 |
| **8** | 9 | 22.523913 | 7.419159 |
| **9** | 10 | 11.398053 | 4.347359 |
| **10** | 11 | 7.497318 | 4.166424 |
| **11** | 12 | 13.138833 | 9.176081 |
| **12** | 13 | 7.771078 | 4.478610 |
| **13** | 14 | 28.762903 | 10.508627 |
| **14** | 15 | 16.075103 | 7.398575 |
| **15** | 16 | 11.060385 | 4.149286 |
| **16** | 17 | 14.037355 | 4.974487 |

Out[14]: (0.0, 54.48671074487035)

**Errors in alignment**

Taken from taLocals

**Tilt series	Error mean (nm)	Error SD (nm)**

**0**	1	0.416	0.289

| **1** | 2 |  | 0.475 | 0.287 |
| --- | --- | --- | --- | --- |
| **2** | 3 |  | 0.370 | 0.286 |
| **3** | 4 |  | 0.366 | 0.270 |
| **4** | 5 |  | 0.426 | 0.277 |
| **5** | 6 |  | 0.391 | 0.262 |
| **6** | 7 |  | 0.388 | 0.272 |
| **7** | 8 |  | 0.434 | 0.296 |
| **8** | 9 |  | 0.361 | 0.264 |
| **9** | 10 |  | 0.398 | 0.279 |
| **10** | 11 |  | 0.406 | 0.279 |
| **11** | 12 |  | 0.400 | 0.264 |
| **12** | 13 |  | 0.426 | 0.286 |
| **13** | 14 |  | 0.468 | 0.298 |
| **14** | 15 |  | 0.483 | 0.296 |
| **15** | 16 |  | 0.365 | 0.259 |
| **16** | 17 |  | 0.345 | 0.269 |

Out[16]: (0.0, 0.81795)

## Tomographic Reconstruction (IMOD)

Reconstruction was performed with IMOD BatchRunTomo.

Reconstruction algorithm: WBP

For more information, please see https://bio3d.colorado.edu/imod/doc/directives.html

Ot2Rec Report for Case Study 2

**Workflow diagram**

# Motion correction

Motion correction has been performed using MotionCor2.

Please cite:

1. Zheng, S., Palovcak, E., Armache, JP. et al.

MotionCor2: anisotropic correction of beam-induced motion for improved cryo-electron microscopy.

*Nat Methods* **14**, 331–332 (2017).

## Summary of shifts for each tilt series

Out[13]:	**count	mean	std	min	25%	50%	75%	max**

**TiltSeries**

**6**	10250.0	0.532437	0.592415	0.0	0.186011	0.358469	0.629682	5.513765

| **7** | 10250.0 | 0.394030 | 0.288621 | 0.0 | 0.189737 | 0.351497 | 0.552268 | 2.871167 |
| --- | --- | --- | --- | --- | --- | --- | --- | --- |
| **9** | 10250.0 | 0.631271 | 0.667577 | 0.0 | 0.226716 | 0.439318 | 0.770114 | 4.961371 |
| **10** | 10250.0 | 0.547538 | 0.402059 | 0.0 | 0.267207 | 0.489591 | 0.758172 | 3.580461 |
| **11** | 10250.0 | 0.289698 | 0.198691 | 0.0 | 0.148661 | 0.269165 | 0.406079 | 1.351481 |
| **12** | 10250.0 | 0.277824 | 0.186826 | 0.0 | 0.141421 | 0.261725 | 0.394462 | 1.213960 |
| **13** | 10250.0 | 0.449222 | 0.545662 | 0.0 | 0.187883 | 0.350143 | 0.566127 | 20.940306 |
| **14** | 10250.0 | 0.344168 | 0.241599 | 0.0 | 0.170880 | 0.314006 | 0.482701 | 1.739224 |
| **15** | 10250.0 | 0.299349 | 0.234410 | 0.0 | 0.141421 | 0.260192 | 0.410000 | 2.072342 |

Out[14]: Text(0.5, 1.0, 'Euclidean Shift Means +/- Standard Deviation')

## Summary of shifts for experiment

Euclidean shifts for all tilt series in the experiment.

# CTF (Defocus) estimation

CTF estimation has been performed using CTFFind4. Please cite:

1. Joseph A. Mindell, Nikolaus Grigorieff. Accurate determination of local defocus and specimen tilt in electron microscopy, *Journal of Structural Biology* **142(3)**:334-347 (2003)
2. Alexis Rohou, Nikolaus Grigorieff. CTFFIND4: Fast and accurate defocus estimation from electron micrographs, *Journal of Structural Biology* **192(2)**:216-221 (2015)

# CTF simulation

CTF simulation has been performed using O2R-ctfsim. Please cite:

1. Alexis Rohou, Nikolaus Grigorieff. CTFFIND4: Fast and accurate defocus estimation from electron micrographs, *Journal of Structural Biology* **192(2)**:216-221 (2015)

# IMOD (general)

IMOD has been used in this image processing operation. Please cite:

1. James R. Kremer, David N. Mastronarde, J.Richard McIntosh. Computer Visualization of ThreeDimensional Image Data Using IMOD, *Journal of Structural Biology* **116(1)**:71-76 (1996)
2. Mastronarde DN, Held SR. Automated tilt series alignment and tomographic reconstruction in IMOD, *Journal of Structural Biology* **197(2)**:102-113 (2017)

## Tilt-series Alignment (IMOD)

## Tomographic Reconstruction (IMOD)

Reconstruction was performed with IMOD BatchRunTomo.

Reconstruction algorithm: WBP

For more information, please see <https://bio3d.colorado.edu/imod/doc/directives.html>

## Richardson-Lucy Deconvolution with RedLionFish

For more information, please see <https://github.com/rosalindfranklininstitute/RedLionfish>

### Deconvolved thumbnails

Ot2Rec Reports for Case Study 3: IMOD aligned data

**Workflow diagram**

# Motion correction

Motion correction has been performed using MotionCor2.

Please cite:

1. Zheng, S., Palovcak, E., Armache, JP. et al. MotionCor2: anisotropic correction of beam-induced motion for improved cryo-electron microscopy. *Nat Methods* **14**, 331–332 (2017).

## Summary of shifts for each tilt series

**mean	std	25%	50%	75%**

**TiltSeries**

**1**	2.056202	2.656386	0.635059	1.234585	2.386598

| **2** | 1.415733 | 1.614418 | 0.521536 | 1.001249 | 1.791368 |
| --- | --- | --- | --- | --- | --- |
| **3** | 1.295799 | 1.117591 | 0.512445 | 0.965660 | 1.759602 |
| **4** | 1.661103 | 2.091689 | 0.577062 | 1.106029 | 1.981691 |
| **5** | 1.298596 | 1.033565 | 0.554437 | 1.041009 | 1.762385 |
| **6** | 1.379971 | 1.221563 | 0.605310 | 1.103087 | 1.829891 |
| **7** | 2.242506 | 2.905982 | 0.682642 | 1.346291 | 2.616104 |
| **8** | 1.402211 | 1.245235 | 0.562228 | 1.070000 | 1.881303 |
| **10** | 1.209009 | 0.884546 | 0.586941 | 1.011929 | 1.598405 |
| **11** | 1.871856 | 2.428340 | 0.586941 | 1.202477 | 2.268127 |
| **12** | 1.623454 | 2.318478 | 0.503289 | 0.973293 | 1.905197 |
| **13** | 6.831695 | 6.379735 | 2.314584 | 5.125159 | 9.487324 |
| **14** | 16.070258 | 49.565171 | 0.590339 | 1.156849 | 2.527969 |
| **15** | 19.491723 | 55.706889 | 0.605392 | 1.180042 | 2.618416 |
| **16** | 24.059852 | 65.467685 | 0.665733 | 1.379021 | 3.073308 |
| **17** | 20.347179 | 59.296513 | 0.617414 | 1.267793 | 2.946994 |
| **18** | 19.982279 | 56.926885 | 0.621772 | 1.191512 | 2.670431 |
| **19** | 23.301804 | 59.528379 | 1.168428 | 2.810970 | 9.441590 |
| **20** | 22.763273 | 73.947772 | 0.609590 | 1.279453 | 3.041772 |
| **21** | 15.023953 | 48.436937 | 0.653911 | 1.341827 | 2.771299 |
| **22** | 12.322676 | 45.376714 | 0.684251 | 1.385135 | 2.668122 |
| **23** | 10.351220 | 35.034275 | 0.687677 | 1.371860 | 2.622532 |
| **24** | 10.273665 | 39.577862 | 0.594643 | 1.135319 | 2.231569 |

Out[12]: Text(0.5, 1.0, 'Euclidean Shift Means +/- Standard Deviation')

# IMOD (general)

IMOD has been used in this image processing operation.

Please cite:

1. James R. Kremer, David N. Mastronarde, J.Richard McIntosh.

Computer Visualization of Three-Dimensional Image Data Using IMOD, *Journal of Structural Biology* **116(1)**:71-76 (1996)

2. Mastronarde DN, Held SR.

Automated tilt series alignment and tomographic reconstruction in IMOD, *Journal of Structural Biology* **197(2)**:102-113 (2017)

## Tilt-series Alignment (IMOD)

### Shifts between patches in A

Taken from .xf file

### Errors in alignment

Taken from taLocals

Out[16]: (0.0, 2.4423)

## Tomographic Reconstruction (IMOD)

Reconstruction was performed with IMOD BatchRunTomo.

Reconstruction algorithm: SIRT

For more information, please see <https://bio3d.colorado.edu/imod/doc/directives.html>

# AreTomo

AreTomo was used in this workflow. Please cite:

1. Shawn Zheng, Georg Wolff, Garrett Greenan, et al. AreTomo: An integrated software package for automated marker-free, motion-corrected cryo-electron tomographic alignment and reconstruction, *JSB: X* **6(100068)** , ISSN 2590-1524, (2022) [DOI](https://doi.org/10.1016/j.yjsbx.2022.100068.) | [ScienceDirect](https://www.sciencedirect.com/science/article/pii/S2590152422000095)

For more information, please see https://msg.ucsf.edu/software.

## Tomographic Reconstruction (AreTomo)

Vol Z = 1000

Sample thickness = -1

Pixel size = 1.47

Reconstruction algorithm = SART

# Savu Reconstruction

Reconstruction was performed with Savu 4.0.

Please cite:

1. Wadeson, N and Basham, M. Savu: A Python-based, MPI Framework for Simultaneous Processing of Multiple, N-dimensional, Large Tomography Datasets. *arXiv* **1610.08015**. (2016)
2. Wadeson, N, Verschoyle, J, Kazantsev, D., et al. DiamondLightSource/Savu: Version 4.0. *Zenodo*.

(2021) [DOI](https://doi.org/10.5281/zenodo.5095360) 10.5281/zenodo.5095360.

3. The HDF Group. Hierarchical Data Format, version 5. (1997-2016)
4. Walker, D and Dongarra, J. MPI: a standard message passing interface. *Supercomputer* **12**: 56-68. (1996)
5. Van Aarle, W, Palenstijn, W, Cant, J, et al. Fast and flexible X-ray tomography using the ASTRA toolbox. *Optics express* **24(22)**: 25129-25147. (2016)
6. Van Aarle, W, Palenstijn, W, De Beenhouwer, J, et al. The ASTRA Toolbox: A platform for advanced algorithm development in electron tomography. *Ultramicroscopy* **157**: 35-47. (2015)
7. Palenstijn, WJ and Batenburg, KJ and Sijbers, J. Performance improvements for iterative electron tomography reconstruction using graphics processing units (GPUs). *JSB* **176(2)** 250-253. (2011) For more information, please see https://savu.readthedocs.io/en/latest/

## Note

Savu was developed by the X-ray tomography community, so the conventional relationship between grey values and density is reversed compared to EM, i.e., the colours are reversed. The visualisations in this report have had their colours inverted to match the EM convention.

Reconstruction algorithm used: CGLS_CUDA

## Tomogram thumbnails

Ot2Rec Report for Case Study 3 – AreTomo aligned data

**Workflow diagram**

# Motion correction

Motion correction has been performed using MotionCor2.

Please cite:

1. Zheng, S., Palovcak, E., Armache, JP. et al. MotionCor2: anisotropic correction of beam-induced motion for improved cryo-electron microscopy. *Nat Methods* **14**, 331–332 (2017).

## Summary of shifts for each tilt series

**mean	std	25%	50%	75%**

**TiltSeries**

**1**	2.056202	2.656386	0.635059	1.234585	2.386598

| **2** | 1.415733 | 1.614418 | 0.521536 | 1.001249 | 1.791368 |
| --- | --- | --- | --- | --- | --- |
| **3** | 1.295799 | 1.117591 | 0.512445 | 0.965660 | 1.759602 |
| **4** | 1.661103 | 2.091689 | 0.577062 | 1.106029 | 1.981691 |
| **5** | 1.298596 | 1.033565 | 0.554437 | 1.041009 | 1.762385 |
| **6** | 1.379971 | 1.221563 | 0.605310 | 1.103087 | 1.829891 |
| **7** | 2.242506 | 2.905982 | 0.682642 | 1.346291 | 2.616104 |
| **8** | 1.402211 | 1.245235 | 0.562228 | 1.070000 | 1.881303 |
| **10** | 1.209009 | 0.884546 | 0.586941 | 1.011929 | 1.598405 |
| **11** | 1.871856 | 2.428340 | 0.586941 | 1.202477 | 2.268127 |
| **12** | 1.623454 | 2.318478 | 0.503289 | 0.973293 | 1.905197 |
| **13** | 6.831695 | 6.379735 | 2.314584 | 5.125159 | 9.487324 |
| **14** | 16.070258 | 49.565171 | 0.590339 | 1.156849 | 2.527969 |
| **15** | 19.491723 | 55.706889 | 0.605392 | 1.180042 | 2.618416 |
| **16** | 24.059852 | 65.467685 | 0.665733 | 1.379021 | 3.073308 |
| **17** | 20.347179 | 59.296513 | 0.617414 | 1.267793 | 2.946994 |
| **18** | 19.982279 | 56.926885 | 0.621772 | 1.191512 | 2.670431 |
| **19** | 23.301804 | 59.528379 | 1.168428 | 2.810970 | 9.441590 |
| **20** | 22.763273 | 73.947772 | 0.609590 | 1.279453 | 3.041772 |
| **21** | 15.023953 | 48.436937 | 0.653911 | 1.341827 | 2.771299 |
| **22** | 12.322676 | 45.376714 | 0.684251 | 1.385135 | 2.668122 |
| **23** | 10.351220 | 35.034275 | 0.687677 | 1.371860 | 2.622532 |
| **24** | 10.273665 | 39.577862 | 0.594643 | 1.135319 | 2.231569 |

Out[12]: Text(0.5, 1.0, 'Euclidean Shift Means +/- Standard Deviation')

# AreTomo

AreTomo was used in this workflow. Please cite:

1. Shawn Zheng, Georg Wolff, Garrett Greenan, et al. AreTomo: An integrated software package for automated marker-free, motion-corrected cryo-electron tomographic alignment and reconstruction, *JSB: X* **6(100068)** , ISSN 2590-1524, (2022) [DOI](https://doi.org/10.1016/j.yjsbx.2022.100068.) | [ScienceDirect](https://www.sciencedirect.com/science/article/pii/S2590152422000095)

For more information, please see [https://msg.ucsf.edu/software.](https://msg.ucsf.edu/software)

## AreTomo Alignment

Out[15]:	**Tilt series	Mean shift (px)	Shift s.d. (px)	Mean shift (A)	Shift s.d. (A)**

**1**	1	249.846455	134.187956	293.819432	157.805036

| **2** | 2 |  | 167.342715 | 105.078591 | 196.795033 | 123.572423 |
| --- | --- | --- | --- | --- | --- | --- |
| **3** | 3 |  | 156.732914 | 85.524085 | 184.317907 | 100.576324 |
| **4** | 4 |  | 257.709137 | 154.558138 | 303.065945 | 181.760370 |
| **5** | 5 |  | 145.165522 | 106.232851 | 170.714654 | 124.929833 |
| **6** | 6 |  | 171.819790 | 121.486764 | 202.060072 | 142.868434 |
| **7** | 7 |  | 316.277157 | 234.309473 | 371.941936 | 275.547940 |
| **8** | 8 |  | 152.049511 | 100.580919 | 178.810225 | 118.283161 |
| **9** | 10 |  | 209.886436 | 145.100036 | 246.826449 | 170.637642 |
| **10** | 11 |  | 191.660644 | 141.893540 | 225.392917 | 166.866803 |
| **11** | 12 |  | 203.012721 | 102.486559 | 238.742960 | 120.524194 |
| **12** | 13 |  | 409.359774 | 357.633795 | 481.407095 | 420.577343 |
| **13** | 14 |  | 169.928989 | 126.169199 | 199.836491 | 148.374978 |
| **14** | 15 |  | 365.881024 | 171.289044 | 430.276084 | 201.435915 |
| **15** | 16 |  | 438.967867 | 237.565736 | 516.226212 | 279.377306 |
| **16** | 17 |  | 300.202655 | 219.939474 | 353.038322 | 258.648822 |
| **17** | 18 |  | 162.764817 | 127.174397 | 191.411424 | 149.557091 |
| **18** | 19 |  | 248.243619 | 341.288754 | 291.934496 | 401.355575 |
| **19** | 20 |  | 267.172073 | 131.402679 | 314.194358 | 154.529551 |
| **20** | 21 |  | 172.336549 | 138.699108 | 202.667781 | 163.110151 |
| **21** | 22 |  | 242.213529 | 178.690878 | 284.843110 | 210.140472 |
| **22** | 23 |  | 330.917261 | 281.295930 | 389.158699 | 330.804013 |
| **23** | 24 |  | 823.669692 | 476.013402 | 968.635558 | 559.791760 |

Out[16]: (0.0, 2000.0)

## Tomographic Reconstruction (AreTomo)

Vol Z = 1000

Sample thickness = -1

Pixel size = 1.47

Reconstruction algorithm = SART

# Savu Reconstruction

Reconstruction was performed with Savu 4.0.

Please cite:

1. Wadeson, N and Basham, M. Savu: A Python-based, MPI Framework for Simultaneous Processing of Multiple, N-dimensional, Large Tomography Datasets. *arXiv* **1610.08015**. (2016)
2. Wadeson, N, Verschoyle, J, Kazantsev, D., et al. DiamondLightSource/Savu: Version 4.0. *Zenodo*.

(2021) [DOI](https://doi.org/10.5281/zenodo.5095360) 10.5281/zenodo.5095360.

3. The HDF Group. Hierarchical Data Format, version 5. (1997-2016)
4. Walker, D and Dongarra, J. MPI: a standard message passing interface. *Supercomputer* **12**: 56-68. (1996)
5. Van Aarle, W, Palenstijn, W, Cant, J, et al. Fast and flexible X-ray tomography using the ASTRA toolbox. *Optics express* **24(22)**: 25129-25147. (2016)
6. Van Aarle, W, Palenstijn, W, De Beenhouwer, J, et al. The ASTRA Toolbox: A platform for advanced algorithm development in electron tomography. *Ultramicroscopy* **157**: 35-47. (2015)
7. Palenstijn, WJ and Batenburg, KJ and Sijbers, J. Performance improvements for iterative electron tomography reconstruction using graphics processing units (GPUs). *JSB* **176(2)** 250-253. (2011) For more information, please see <https://savu.readthedocs.io/en/latest/>

## Note

Savu was developed by the X-ray tomography community, so the conventional relationship between grey values and density is reversed compared to EM, i.e., the colours are reversed. The visualisations in this report have had their colours inverted to match the EM convention.

Reconstruction algorithm used: CGLS_CUDA

## Tomogram thumbnails

Table S1: Configuration parameters for Case Study 1.

| Motion correction | |
| --- | --- |
| Input filetype | MRC |
| Gain reference | No gain |
| Pixel size (A) | 2.243 |
| Desired pixel size (A) | 2.243 |
| Discard frames | None |
| Tolerance | 0.5 |
| Max number of iterations | 10 |
| Patch size | 5 x 5 x 20 |
| Use subgroups | Yes |
| IMOD Alignment | |
| Excluded views | None |
| Use rawtlt | Yes |
| Pixel size (nm) | 0.2243 |
| Rotation angle (deg) | 175.51 |
| Stack bin factor | 2 |
| Remove x-rays | Yes |
| Coarse align | |
| Patch size | 210 x 203 |
| Number of patches | 24 x 24 |
| Number of iterations | 4 |
| Limits on shifts | 2, 2 |
| Adjust tilt angles | Yes |
| Fine align | |
| Number of surfaces | 1 |
| Magnification option | fixed |
| Tilt option | fixed |
| Rotation option | group |
| Beam tilt option | fixed |
| Use robust fitting | Yes |
| Weight all contours | Yes |
| IMOD Reconstruction | |
| Use rawtlt | Yes |
| Pixel size (nm) | 0.4486 |
| Rotation angle (deg) | 175.51 |
| Do positioning | No |
| Unbinned thickness | 1920 |
| Correct CTF | No |
| Erase gold | No |
| 2D filtering | No |
| Bin factor | 1 |
| Reconstruction thickness | 1920 |
| Algorithm | WBP |
| Trim volume | Yes |
| Trim volume reorientation | Rotate |

Table S2: Configuration parameters for Case Study 2

| Motion correction | |
| --- | --- |
| Input filetype | MRC |
| Gain reference | No gain |
| Pixel size (A) | 1.87 |
| Desired pixel size (A) | 1.87 |
| Discard frames | None |
| Tolerance | 0.5 |
| Max number of iterations | 10 |
| Patch size | 5 x 5 x 20 |
| Use subgroups | Yes |
| IMOD Alignment | |
| Excluded views | None |
| Use rawtlt | Yes |
| Pixel size (nm) | 0.187 |
| Rotation angle (deg) | -83.96 |
| Stack bin factor | 8 |
| Remove x-rays | Yes |
| Coarse align | |
| Patch size | 224 x 224 |
| Number of patches | 24 x 24 |
| Number of iterations | 4 |
| Limits on shifts | 2, 2 |
| Adjust tilt angles | Yes |
| Fine align | |
| Number of surfaces | 1 |
| Magnification option | Fixed |
| Tilt option | Fixed |
| Rotation option | Group |
| Beam tilt option | Fixed |
| Use robust fitting | Yes |
| Weight all contours | Yes |
| IMOD Reconstruction | |
| Use rawtlt | Yes |
| Pixel size (nm) | 1.496 |
| Rotation angle (deg) | -83.96 |
| Do positioning | No |
| Unbinned thickness | 4096 |
| Correct CTF | No |
| Erase gold | No |
| 2D filtering | No |
| Bin factor | 8 |
| Reconstruction thickness | 4096 |
| Algorithm | WBP |
| Trim volume | Yes |
| Trim volume reorientation | Rotate |
| CTFFind4 | |
| Pixel size (A) | 1.87 |
| Voltage (kV) | 300 |
| Spherical aberration | 2.7 |
| Amp contrast | 0.8 |
| Amp spec size | 512 |
| Min resolution | 30 |
| Max resolution | 5 |
| Defocus min | 5000 |
| Defocus max | 50000 |
| Defocus step | 500 |
| Astigmatism type | None |
| Exhaustive search | No |
| Astigmatism restraint | No |
| Phase shift | No |

Table S3: Configuration parameters for Case Study 3

| Motion correction | |
| --- | --- |
| Input filetype | EER |
| Gain reference | No gain |
| Pixel size (A) | 1.47 |
| Desired pixel size (A) | 1.47 |
| Discard frames | None |
| Tolerance | 0.5 |
| Max number of iterations | 10 |
| Patch size | 5 x 5 x 20 |
| Use subgroups | Yes |
| IMOD Alignment | |
| Excluded views | None |
| Use rawtlt | Yes |
| Pixel size (nm) | 0.147 |
| Rotation angle (deg) | -84.22 |
| Stack bin factor | 8 |
| Remove x-rays | Yes |
| Coarse align | |
| Patch size | 224 x 224 |
| Number of patches | 24 x 24 |
| Number of iterations | 4 |
| Limits on shifts | 2, 2 |
| Adjust tilt angles | Yes |
| Fine align | |
| Number of surfaces | 1 |
| Magnification option | Fixed |
| Tilt option | Fixed |
| Rotation option | Group |
| Beam tilt option | Fixed |
| Use robust fitting | Yes |
| Weight all contours | Yes |
| IMOD Reconstruction | |
| Use rawtlt | Yes |
| Pixel size (nm) | 1.176 |
| Rotation angle (deg) | -84.22 |
| Do positioning | No |
| Unbinned thickness | 512 |
| Correct CTF | No |
| Erase gold | No |
| 2D filtering | No |
| Bin factor | 8 |
| Reconstruction thickness | 4096 |
| Algorithm | SIRT |
| SIRT iterations | 10 |
| Trim volume | Yes |
| Trim volume reorientation | Rotate |
| AreTomo alignment | |
| Bin factor | 8 |
| AreTomo reconstruction | |
| Rotation angle | -84.22 |
| Bin factor | 1 |
| VolZ | 1000 |
| Pixel size (A) | 1.47 |
| Algorithm | SART |
| Savu reconstruction | |
| Algorithm | CGLS |
| Number of iterations | 100 |
| Centre of rotation | 256 |

Guide to writing plugins for Ot2Rec, available at https://github.com/rosalindfranklininstitute/Ot2Rec/wiki/Tutorial:-How-to-write-an-Ot2Rec-plugin

# Guide to writing plugins for Ot2Rec v0.2.x

## Introduction

Plugins in Ot2Rec achieve four main objectives:

1. Capture user arguments for the task. (These are saved in a yaml file)
2. Generate the correct commands to call other programs to complete the task, e.g. IMOD
3. Save the metadata along the way
4. (Optionally) add a section to the report showing how the task performed.

This guide will show you how to write an Ot2Rec plugin called plugin.

## To-Do list

- Add the user arguments to magicgui.py
- Add the desired layout of plugin.yaml to params.py
- Create plugin.py, which holds most of the logic behind your new plugin.
- Add your command-line binding to setup.py. This binding allows users to call your plugin straight from the terminal.

## Step 0: Setup your conda environment

Clone Ot2Rec from Github:

git clone https://github.com/rosalindfranklininstitute/Ot2Rec.git

Create a new conda environment and install Ot2Rec in editable mode:

conda create -n ot2rec

conda activate ot2rec

pip install -e .

This should update your installation of Ot2Rec when changes are made so you are always running the latest version.

## Step 1: Capture user arguments

We use [magicgui](https://napari.org/magicgui/) to generate Ot2Rec's GUI to capture user arguments. These argument capture functions are added to the magicgui.py file. One GUI is created for each plugin, an example is shown below:

**add image here**

Here is a minimal example of the magicgui functions we need for our plugin.

```
@mg(
    call_button="Create config file",
    layout="vertical",
    result_widget=False,

    project_name={"label": "Project name *"},
    pixel_size={"label": "Pixel size in A",
                "min": 0.001,
    },
    rootname={"label": "Rootname of current project (required if different from project name"},
    suffix={"label": "Suffix of project files"},
    input_mrc_folder={
        "label": "Folder containing input mrc's",
        "mode": "d",
    },
    output_path={
        "label": "Path to output folder",
        "mode": "d",
    },
    choice_abc={
        "label": "Choice between a, b, c",
        "choices": ["a", "b", "c]
    },
)
def get_args_plugin(
        project_name="",
        pixel_size=0.00,
        rootname="",
        suffix="",
        input_mrc_folder=Path("./plugin_input"),
        output_path=Path("./plugin_output"),
        recon_algo="WBP",
):
    return locals()
```

First, we use the magicgui decorator @mg to create our widgets. The widgets we have on all our plugins are:

- project_name | name of the dataset we are processing
- rootname | prefix of all the files we want to work with, defaults to the project_name if not specified.
- suffix | suffix added to the end of the filenames, can be left as None.
- some sort of input, e.g., input_mrc_folder
- an output path, e.g., output_path

## Step 2: Create template of yaml to hold arguments

The plugin.yaml file holds the information needed to set up the task to be performed, e.g., input filepaths, parameters, output filepaths. Some of these are captured from the magicgui in the previous section, but others can be calculated or populated with default values.

The yaml file is first generated from a template in params.py, its values are then populated by code in plugin.py with user arguments captured from magicgui.py.

An example yaml file for our plugin looks like:

```
System:
    process_list:
    - 1
    - 2
    - 3
    output_path: ./plugin_processed
    output_rootname: neurone
    output_suffix: ''
Plugin_setup:
    pixel_size: 1.0
    input_mrc:
    - ./aligned/neurone_0001/neurone_0001.st
    - ./aligned/neurone_0002/neurone_0002.st
    - ./aligned/neurone_0003/neurone_0003.st
    output_mrc:
    - ./aligned/neurone_0001/neurone_0001_ali.mrc
    - ./aligned/neurone_0002/neurone_0002_ali.mrc
    - ./aligned/neurone_0003/neurone_0003_ali.mrc
    tilt_angles:
    - ./aligned/neurone_0001/neurone_0001.rawtlt
    - ./aligned/neurone_0002/neurone_0002.rawtlt
    - ./aligned/neurone_0003/neurone_0003.rawtlt
    choice: a
```

Here, the process_list, input_mrc, output_mrc, and tilt_angles fields are populated automatically in the plugin.update_yaml function in plugin.py.

To create the template plugin.yaml file, we add the following to params.py:

```
def new_plugin_yaml(args):
    """
    Subroutine to create yaml file for plugin

    ARGS:
    args (Namespace) :: Namespace containing user parameter inputs
    """

    plugin_yaml_name = args.project_name.value + '_plugin.yaml'

    plugin_yaml_dict = {
        'System': {
            'process_list': None,
            'output_path': str(args.output_path.value),
            'output_rootname': args.project_name.value if args.rootname.value is None else args.rootname.value,
            'output_suffix': args.suffix.value,
        },

        'Plugin_setup': {
            'pixel_size': args.pixel_size.value,
            'input_mrc': None,
            'output_mrc': None,
            'tilt_angles': None,
            'choice': args.choice.value,
        }
    }

    with open(plugin_yaml_name, 'w') as f:
        yaml.dump(plugin_yaml_dict, f, indent=4, sort_keys=False)
```

Don't worry about populating all the fields just now, just fill in those which can be obtained from the args, i.e., the user arguments captured from magicgui.

Next, we will need to create the plugin.py file, which will hold most of the logic needed for the plugin. We will go through this in two parts, here we will write the sections which deal with creating the yaml to setup the plugin.

Add the create_yaml and update_yaml methods to plugin.py.

```
def update_yaml(args):
    """Method to update yaml file

    Here we set the process list, specific filepaths, and check inputs

    ARGS:
    args (magicgui.FunctionGUI) :: magicgui object containing user input
    """
    # Read in template yaml file
    plugin_yaml_name = f"{args["project_name"]}_plugin.yaml"
    plugin_params = prmMod.read_yaml(
        project_name=args["project_name"],
        filename=plugin_yaml_name
    )

    # Set input filepaths, can be by searching for specific filenames in the input mrc folder, then update the yaml file
    input_files = glob(f"{args['input_mrc']}/*.mrc")
    plugin_params.params["Plugin_setup"]["input_mrc"] = input_files

    # Can do similar for the output filepaths

    # Set process list, this is usually the tilt series index, can be parsed from filepaths

    # Update yaml
    with open(Path(plugin_yaml_name), "w") as f:
        yaml.dump(plugin_params.params, f, indent=4, sort_keys=False)

def create_yaml(input_mgNS=None):
    """
    Subroutine to create new yaml file for Plugin
    """

    # Parse user inputs
    if input_mgNS is None:
        args = mgMod.get_args_plugin.show(run=True).asdict()
    else:
        args = input_mgNS

    # Create the yaml file, then automatically update it
    prmMod.new_plugin_yaml(args)
    update_yaml(args)
```

Now the functions to create the yaml should be in place, and we can move on to generating and running commands based on the yaml file.

## Step 3: Functions to do plugin's work

The Plugin class in plugin.py creates the results folders, generates commands to run the plugin, runs the plugin, and saves the metadata.

Let's write the Plugin class in stages. First, set up the Plugin class. All Ot2Rec plugin classes have the same fundamental attributes

- project_name
- params_in: parameters read from the yaml file
- logger_in: a logger object to create the logfile o2r_plugin.log
- md_out: dictionary of metadata to pass to plugin_mdout.yaml.

In the __init__, we also want to set up the results folder structure, which is done in the _get_internal_metadata() function.

```
class Plugin:
    def __init__(self, project_name, params_in, logger_in):
        self.proj_name = project_name
        self.params = params_in.params
        self.logObj = logger_in
        self.md_out = {}

        self._get_internal_metadata()

    def _get_internal_metadata(self):
        """Prep internal metadata for processing and checking

        ** See other plugins for code to reuse **

        Generally, this sets the input and output filepaths and creates results folders.

        You can also pass information to the md_out, which is a metadata yaml file written after processing. e.g., putting in filepaths to the results.
        """
        for curr_ts in self.params["System"]["process_list"]:
            subfolder = (f"{self.basis_folder}/"
                         f"{self.rootname}_{curr_ts:04d}{self.suffix}")
            os.makedirs(subfolder, exist_ok=True)

            self.md_out["plugin_output_dir"][curr_ts] = subfolder
            self.md_out["plugin_output_file"][curr_ts] = f"{subfolder}/example.st
```

Next, we want to generate the commands needed to run the plugin. These are the same commands we would use if we were to use the plugin program directly (e.g., IMOD, AreTomo) in the terminal.

Add the following to the Plugin class.

```
    def _get_plugin_command(self, i):
        """Get command to call an external process for the i-th tilt series"""
        cmd = [
            "plugin",
            "-input",
            self.params["Plugin_setup"]["input_mrc"][i],
            "-output",
            self.params["Plugin_setup"]["output_mrc"][i],
            "-tiltangle",
            self.params["Plugin_setup"]["tilt_angles"][i],
            "-choice",
            self.params["Plugin_setup"]["choice]
        ]

        return cmd
```

We also want to add the runner functions to the Plugin class.

```
    def _run_plugin(self, i):
        """Run the plugin for the i-th tilt series"""
        cmd = self._get_plugin_command(i)
        plugin_run = subprocess.run(
            cmd,
            stdout=subprocess.PIPE,
            stderr=subprocess.STDOUT,
            encoding="ascii",
            check=True
        )
        self.logObj(plugin_run.stdout) # save stdout to log

    def run_plugin_all(self):
        for i, ts in enumerate(self.params["System"]["process_list"]):
            self._run_plugin(i)
        self.export_metadata()

    def export_metadata(self):
        yaml_file = self.proj_name + "_plugin_mdout.yaml"
        with open(yaml_file, "w") as f:
            yaml.dump(self.md_out, f, indent=4, sort_keys=False)
```

Lastly, we will add the run function to plugin.py, which is called by o2r.plugin.run.

```
def run():
    """
    Method to run plugin
    """
    # argparser to collect the project name
    parser = argparse.ArgumentParser()
    parser.add_argument("project_name",
                        type=str,
                        help="Name of current project")
    args = parser.parse_args()

    # Check if prerequisite files exist
    plugin_yaml_name = f"{args["project_name"]}_plugin.yaml"
    if not os.path.isfile(plugin_yaml_name):
        raise IOError("Error in Ot2Rec.main.run_plugin: plugin yaml file not found.")

    # Read in config and metadata
    plugin_config = prmMod.read_yaml(
        project_name=args.project_name,
        filename=plugin_yaml_name
    )

    # Create Logger object
    logger = logMod.Logger(log_path="o2r_plugin.log")

    # Create Plugin object
    plugin_obj = Plugin(
        project_name=args.project_name,
        params_in=plugin_config,
        logger_in=logger
    )

    # Run AreTomo commands
    plugin_obj.run_plugin_all()
```

## Step 4: Add CLI binding

Lastly, add the plugin's entry points to setup.py to use it in the command line. In setup.py under entry_points, add:

"o2r.plugin.new=Ot2Rec.plugin:create_yaml",

"o2r.plugin.run=Ot2Rec.plugin:run",

## Step 5: Share your plugin

If you'd like your plugin to be part of the public Ot2Rec, raise a pull request and we'll review your code and merge it if everything works well.

Tutorial to reproduce Case Study 1, available from <https://github.com/rosalindfranklininstitute/Ot2Rec/wiki/Tutorial:-Basic-reconstruction-workflow-in-Ot2Rec>

This tutorial covers tomogram reconstruction of EMPIAR 10364 with Ot2Rec. Here we’ve assumed that Ot2Rec, Ot2Rec Report, MotionCor2 and IMOD are already installed on your system. If you need help with this, please see the Ot2Rec / Ot2Rec Report wikis, the MotionCor2 installation page, or the IMOD installation page. You’ll need to run this on a GPU enabled Linux system. For reference, our system we used for this tutorial was a virtual Linux machine with 12 Intel Xeon Gold 5218 2.30GHz CPUs and 1 Tesla V100 GP.

1. Download the [example data](https://www.ebi.ac.uk/empiar/EMPIAR-10364/)
2. Open a terminal and navigate to where the data is stored (replace /path/to/data with the actual path)

cd /path/to/data

3. Create a new folder – this will be where your processing will be stored.

mkdir processing

cd processing

4. Start a new Ot2Rec project by running

o2r.new

This creates a new Ot2Rec project and brings up the GUI to fill in the required parameters.

For this dataset, an example image filename is:

Click on create config file and close the GUI. There will now be 3 new files created:

- new_proj.yaml – a logfile capturing output from Ot2Rec
- TS_proj.yaml – a metadata file showing the parameters you’ve just filled into the GUI
- TS_master_md.yaml – this holds all a record of the tilt angles, filenames, tilt series number, and image number, which have been extracted from the filenames. Ot2Rec uses this information to generate the commands to run processes on all the tilt series.

5. We will now set up the Ot2Rec motioncor2 plugin.

First, ensure that motioncor2 is available on your system (RFI users: use module load motioncor2)

o2r.mc.new

Fill in the project name and pixel size of the data. The pixel size can usually be found in the mdoc or in the mrc header of your images. The MC2 output folder field sets where the motion corrected images will be stored, in this case, in the motioncor folder. You can change this if you’d like to. We have left the remaining parameters at their default values but you are free to change them if you would like to. Note that the path to mc2 executable field will probably be specific to your institution or system, depending on how motioncor2 is installed.

Again, click on the create config file button, close the dialog and two files will be created in your processing folder.

- o2r_motioncor2.log – this stores the output of motioncor which appears in the terminal.
- TS_mc2.yaml – this stores the user input you’ve added earlier, along with a process list.

The process list is the list of tilt series that will be processed if we run this plugin. You can edit this manually to only use a selection of the tilt series if you would like to.

6. To run the motioncor2 plugin, we run

o2r.mc.run TS

Running Ot2Rec plugins requires the project name to be specified, so in this case we add “TS” after the run command to tell Ot2Rec to run the motion correction plugin on the project TS.

This will likely take a while but you can monitor the progress in the terminal. When complete, your motioncor folder should look like this:

7. Now we will set up the tilt series alignment in IMOD.

o2r.imod.align.new

Fill in the relevant parameters, referring to the mdoc if necessary. Only parameters with *’s are compulsory to fill in.

Again, create the config file and close the dialog box. Two files will be created:

- o2r_imod_align.log – holds the output from IMOD’s alignment method
- TS_align.log – holds the configuration parameters from the input you just filled in.

Note that you may have to change the Path to BatchRunTomo directives template depending on where IMOD is installed on your system.

8. Run the alignment step. Ensure IMOD is loaded (RFI users: use module load imod) Run the Ot2Rec IMOD alignment plugin:

o2r.imod.align.run TS

IMOD will then create the stacks (.st files) from the motion corrected files, using the filepaths saved in the TS_mc2_mdout.yaml file from step 6. After that, alignment will start. You can monitor the process in the terminal. If you’d like to view your aligned tilt series, you can open the _ali.mrc files in IMOD’s 3dmod or in Fiji.

9. Set up the IMOD reconstruction process.

o2r.imod.recon.new

The thickness parameters determine how many z-slices will be in the reconstruction. Larger thickness values means you are less likely to inadvertently exclude regions of your specimen in the tomogram, but there might be lots of empty space in the tomogram. You can experiment with these parameters by doing a test run – generate the TS_recon.yaml file and edit the process list to only do one tilt series and see if you are happy with the result. Once you are, then you can generate a new TS_recon.yaml by rerunning this step with the parameters you have chosen.

10. Run the IMOD reconstruction process

o2r.imod.recon.run TS

11. Generate the Ot2Rec report. (temporary fix – this step will be redundant in the future)

Run o2r.imod.align.stats to generate the statistics for the alignment step to go into the report.

Activate the Ot2Rec report environment (see the Github for detailed instructions if required).

Generate the report by running:

o2r.report.run TS --to_html --to_slides

This generates the report as a html document that you can print to a pdf, or to a slide deck that you can also open in the browser. The report will be in your processing folder and it will be called “report.html” or “report.slides.html”.

**import** Ot2Rec **as** o2r **import** magicgui **import** yaml

In [1]:

**Example: Using Ot2Rec in Jupyter Notebooks**

We will be processing [EMPIAR-10364](https://www.ebi.ac.uk/empiar/EMPIAR-10364/) in this example. You will also need [Ot2Rec](https://github.com/rosalindfranklininstitute/Ot2Rec), [IMOD,](https://bio3d.colorado.edu/imod/) and [MotionCor2](https://emcore.ucsf.edu/ucsf-software) installed. You'll need to run this on a GPU enabled Linux system. For reference, our system is a virtual Linux machine with 12 Intel Xeon Gold 5218 2.30 GHz CPUs and 1 Tesla V100 GP.

**1. Download the data**

Download the data from [EMPIAR-10364](https://www.ebi.ac.uk/empiar/EMPIAR-10364/).

# 2. Create a new Ot2Rec project

The next code cell will bring up a MagicGUI dialogue box where you can fill in your input.

o2r**.**main**.**new_proj()

In [3]:

Master metadata file created.

Alternatively, if you prefer to set the user inputs in Jupyter directly, you can create the input arguments directly and "deconstruct" the o2r.main.new_proj function.

*# Set user args, look at o2r.magicgui for names of fields etc*

new_proj_args **=** magicgui**.**widgets**.**FunctionGui(o2r**.**magicgui**.**get_args_new_proj) new_proj_args**.**project_name**.**value **=** "TS" new_proj_args**.**source_folder**.**value **=** "../data" new_proj_args**.**no_mdoc print(new_proj_args)

*# Create main yaml config file*

o2r**.**params**.**new_master_yaml(new_proj_args)

*# Create new empty metadata object and turn it into a yaml* meta **=** o2r**.**metadata**.**Metadata( project_name**=**"TS", job_type**=**"master"

)

meta**.**create_master_metadata()

**with** open("TS_master_md.yaml", "w") **as** f: yaml**.**dump(meta**.**metadata, f, indent**=**4)

In [10]:

<FunctionGui magicgui.widgets._function_gui.<class 'magicgui.widgets._function_gu

i.FunctionGui'>(project_name='TS', source_folder=PosixPath('/ceph/groups/els/EMPIA R-10364/example/../data'), folder_prefix='', file_prefix='', ext='mrc', stack_fiel d=0, index_field=1, tiltangle_field=2, no_mdoc=True)>

A yaml file containing the setup details for the project has now been created.

**3. Run MotionCor2.**

Ensure motioncor2 is available on your system for this step. Again, you can choose to enter your user inputs in magicgui, or set them directly. Both ways are included below.

In [14]: *# Use MagicGUI to capture inputs* o2r**.**motioncorr**.**create_yaml()

Master config read successfully.

Master metadata read successfully.

Previous MotionCor2 metadata not found.

MotionCor2 metadata updated.

MotionCor2 metadata file created.

In [15]: *# *OR* set user args manually* mc2_args **=** magicgui**.**widgets**.**FunctionGui(o2r**.**magicgui**.**get_args_mc2) mc2_args**.**project_name**.**value **=** "TS" mc2_args**.**pixel_size**.**value **=** 2.243 o2r**.**motioncorr**.**create_yaml(mc2_args) *# see tests for more examples of setting the u*

Master config read successfully.

Master metadata read successfully.

Previous MotionCor2 metadata not found.

MotionCor2 metadata updated.

MotionCor2 metadata file created.

Now we will run motioncor2. Since this is an example, I have edited the TS_mc2.yaml process list to only process one tilt-series, but you can leave the process list as is if you'd like. The following code cell is a "deconstructed" version of what happens when you call o2r.mc.run in the terminal.

```
mc2_params = o2r.params.read_yaml(     project_name="TS",     filename="TS_mc2.yaml",
)
proj_md = o2r.metadata.read_md_yaml(     project_name="TS",     job_type="motioncorr",     filename="TS_master_md.yaml",
)
mc2_obj = o2r.motioncorr.Motioncorr(     project_name="TS",     mc2_params=mc2_params,     md_in=proj_md,     logger=o2r.logger.Logger("o2r_motioncor2.log")
)
mc2_obj.run_mc2() mc2_obj.export_metadata()
```

In [18]:

Ot2Rec-MotionCor2 started.

Processing TS 1...: 100%|████████████████████████████████████████████| 1/1 [02:34<

00:00, 154.23s/it]

Ot2Rec-MotionCor2 jobs finished.

# 4. Run IMOD alignment

Ensure IMOD is available on your system. User inputs can be captured from the MagicGUI or programatically as before.

*# MagicGUI method* o2r**.**align**.**create_yaml()

In [20]:

```
# Programatically
align_args = magicgui.widgets.FunctionGui(o2r.magicgui.get_args_align) align_args.project_name.value = "TS" align_args.rot_angle.value = 175.51
align_args.stack_bin_factor.value = 8 # to speed things up o2r.align.create_yaml(align_args)
```

In [2]:

```
# Run
align_params = o2r.params.read_yaml(     project_name="TS",     filename="TS_align.yaml",
)
mc2_md = o2r.metadata.read_md_yaml(     project_name="TS",     job_type="align",     filename="TS_mc2_mdout.yaml"
)
align_obj = o2r.align.Align(     project_name="TS",     md_in=mc2_md,     params_in=align_params,     logger_in=o2r.logger.Logger("o2r_imod_align.log"),
)
align_obj.create_stack_folders() align_obj.create_rawtlt() align_obj.create_stack() align_obj.align_stack()
```

In [2]:

Ot2Rec-align (IMOD) started: newstack.

Creating stack for TS 1...: 100%|█████████████████████████████████████| 1/1 [00:06

<00:00, 6.17s/it]

All Ot2Rec-align (IMOD): newstack jobs successfully finished.

Ot2Rec-align (IMOD) started: batchruntomo.

Aligning TS 1...: 100%|██████████████████████████████████████████████| 1/1 [03:37<

00:00, 217.02s/it]

All Ot2Rec-align (IMOD) jobs successfully finished.

# 4. Reconstruction with IMOD

*# Create yaml* o2r**.**recon**.**create_yaml()

In [3]:

IMOD alignment metadata read successfully.

Previous IMOD reconstruction metadata not found.

IMOD reconstruction metadata updated.

IMOD alignment metadata file created.

In [4]: *# Programatically* recon_args **=** magicgui**.**widgets**.**FunctionGui(o2r**.**magicgui**.**get_args_recon) recon_args**.**project_name**.**value **=** "TS" o2r**.**recon**.**create_yaml(recon_args)

IMOD alignment metadata read successfully.

Previous IMOD reconstruction metadata not found.

IMOD reconstruction metadata updated.

IMOD alignment metadata file created.

```
# Run
recon_params = o2r.params.read_yaml(     project_name="TS",     filename="TS_recon.yaml",
)
align_md = o2r.metadata.read_md_yaml(     project_name="TS",     job_type="reconstruct",     filename="TS_align_mdout.yaml"
)
recon_obj = o2r.recon.Recon(     project_name="TS",     md_in=align_md,     params_in=recon_params,     logger_in=o2r.logger.Logger("o2r.imod_recon.log"),
) recon_obj.recon_stack()
```

In [2]:

Ot2Rec-reconstruction (IMOD) started.

Reconstructing TS 1...: 100%|████████████████████████████████████████| 1/1 [03:49<

00:00, 229.67s/it]

All Ot2Rec-recon (IMOD) jobs successfully finished.

# 5. Ot2Rec reports

You can generate the Ot2Rec reports by running o2r.report.run TS in the project directory within a conda env with ot2rec_report installed.

Movie S1: Video of 3D reconstructions from the various workflows in Case Study 3

Full animation available at DOI: 10.6084/m9.figshare.21732491

IMOD Alignment	AreTomo Alignment

IMOD SIRT

AreTomo SART

Savu CGLS
